# The Influence of Portal Deviation on the Effect of Repeat Dearterializations of a Transplantable Adenocarcinoma to the Rat Liver

**DOI:** 10.1155/1994/68212

**Published:** 1994

**Authors:** L. Q. Wang, B. G. Persson, S. Bengmark

**Affiliations:** Department of Surgery Lund University Lund Sweden

## Abstract

As liver tumours receive some of their blood supply from the portal vein, we wanted to illustrate the influence of portal blood flow in combination with dearterialization in the treatment of liver tumours. Forty male, inbred Wistar/Furth rats with an adenocarcinoma transplanted to the liver were treated with various inflow occlusions repeated daily for 5 days. Deviation of the portal blood flow alone with an end-side porto-caval shunt did not alter the tumour growth (*p* = 0.089). Thirty min of repeat dearterializations was potentiated by portal deviation so that tumour growth was delayed (*p* = 0.004). However, repeat dearterializations for 60 min in portal deviated rats induced irreversible liver damage and all rats died in a few days. Repeated dearterializations for 60 minutes alone retarded the tumour growth as efficiently (*p* = 0.007). Simultaneous occlusion of the hepatic artery and the portal vein for 30 minutes with a side-side porto-caval shunted (total devascularization) did not affect tumour growth (*p* = 0.154). Liver aminotransferases (ASAT and ALAT) were substantially increased following dearterialization for 30 min in rats with either an end-side or a side-side porto-caval shunt. Dearterialization for 60 min in rats with end-side porto-caval shunts gave a further release of ASAT and ALAT.

In conclusion, portal deviation did not augment the therapeutic benefit of repeat dearterializations for the treatment of this experimental liver tumour. Repeat dearterializations alone seemed to be a feasible and efficient therapy for liver tumours.


*HPB Surgery*, 1994, Vol. 8, pp. 37–41
Reprints available directly from the publisher
Photocopying permitted by license only

© 1994 Harwood Academic Publishers GmbH
Printed in Malaysia

### The Influence of Portal Deviation on the Effect of Repeat Dearterializations of a Transplantable Adenocarcinoma to the Rat Liver

L. Q. WANG, B. G. PERSSON and S. BENGMARK

Department of Surgery, Lund University, Lund, Sweden

As liver tumours receive some of their blood supply from the portal vein, we wanted to illustrate the influence of portal blood flow in combination with dearterialization in the treatment of liver tumours. Forty male, inbred Wistar/Furth rats with an adenocarcinoma transplanted to the liver were treated with various inflow occlusions repeated daily for 5 days. Deviation of the portal blood flow alone with an end-side porto-caval shunt did not alter the tumour growth ($p = 0.089$). Thirty min of repeat dearterializations was potentiated by portal deviation so that tumour growth was delayed ($p = 0.004$). However, repeat dearterializations for 60 min in portal deviated rats induced irreversible liver damage and all rats died in a few days. Repeated dearterializations for 60 minutes alone retarded the tumour growth as efficiently ($p = 0.007$). Simultaneous occlusion of the hepatic artery and the portal vein for 30 minutes with a side-side porto-caval shunted (total devascularization) did not affect tumour growth ($p = 0.154$). Liver aminotransferases (ASAT and ALAT) were substantially increased following dearterialization for 30 min in rats with either an end-side or a side-side porto-caval shunt. Dearterialization for 60 min in rats with end-side porto-caval shunts gave a further release of ASAT and ALAT.

In conclusion, portal deviation did not augment the therapeutic benefit of repeat dearterializations for the treatment of this experimental liver tumour. Repeat dearterializations alone seemed to be a feasible and efficient therapy for liver tumours.

KEY WORDS: Ischemia dearterialization liver tumour porto-caval shunt

### INTRODUCTION

The theory that established liver tumours are predominantly supplied from the hepatic artery[1–4] has led to extensive use of hepatic artery ligation (HAL), dearterialization and cytotoxic perfusion through the hepatic artery in the treatment of both primary and secondary malignant liver tumours. Some studies have shown that small liver tumours and tumour cells in the peripheral zone are supplied from both the hepatic artery and the portal vein[5,6]. Intra portal chemotherapy has been tried as portal blood flow has been shown to significantly increase following HAL[7]. Furthermore, a favourable response to repeat dearterializations was observed in hepatocellular carcinoma (HCC) patients with an obstructed portal vein[8] suggesting that portal blood flow to the tumour might play a role during ischemic treatment of liver tumours.

This experiment was designed to find out whether occlusion of both portal and hepatic arterial blood flow was more efficient in retarding the growth of a liver tumour than either alone and also to study the injurious effects of such procedures on the normal liver. A porto-caval shunt was used to deprive the tumour of its portal supply and the hepatic artery was intermittently and briefly occluded with an implantable occluder as described previously[9].

*Address for correspondence*: Bo G. Persson, MD. Ph.D., Department of Surgery, Lund University, S-221 85 LUND, Sweden.

### MATERIALS AND METHODS

Male inbred Wistar/Furth (W/F) rats weighing 200–300 g were used. They were housed three per cage and fed standard laboratory diet ad libitum. The tumour used was a *N*-methyl-*N*-nitrosoguanidine-induced colonic adenocarcinoma propagated intraperitoneally by weekly injections[10]. The number of passages before the generation used in this study was[30]. Under chloral-hydrate (0.25 g/kg; Apoteksbolaget, Stockholm, Sweden) anaesthesia the abdominal cavity was opened through a midline incision and a suspension containing $1 \times 10^6$ viable tumour cells was injected subcapsularly into the left lobe of the liver. Five days after inoculation the tumours were measured with callipers and the rats randomly allocated to the various treatment groups. Creation of a porto-caval shunt (end-side or side-side) and implantation of the occluder were performed at the same laparotomy. On the sixth day after treatment all rats were killed with ether, the tumours remeasured and their volumes calculated according to the formula: $V = axb^2/2$ ($a =$ the longest and $b =$ the shortest diameter of the tumour)[11].

Forty rats were randomly assigned to one of 7 groups:

Group A: Sham-operated control ($n = 6$);
Group B: Deviation of portal blood flow with an end-side portacaval shunt ($n = 6$);
Group C: Repeated simultaneous occlusion of both the hepatic artery and the portal vein (total devascularization) for 30 minutes daily with a side-side portacaval shunt ($n = 6$);
Group D: Repeated dearterializations for 30 minutes daily with an end-side portacaval shunt ($n = 6$);
Group E: Repeated dearterializations for 60 minutes daily with an end-side portacaval shunt ($n = 4$);
Group F: Repeated dearterializations for 30 minutes daily ($n = 6$);
Group G: Repeated dearterializations for 60 minutes daily ($n = 6$).

The surgical procedures were as follows. The portal vein was mobilized from the hepatic artery and transected as close to the hilum as possible. The inferior vena cava was then mobilized just above the right renal vein without posterior dissection. An angled vascular clamp was placed across a segment of the anteromedial wall of the inferior vena cava and an end-side porto-caval anastomosis was created with 8/0 silk. A side-side porto-caval anastomosis was created after mobilization of the portal vein and the inferior vena cava. The inferior vena cava was entirely freed from its surroundings above the right renal vein up to the liver and the portal vein likewise isolated to prevent tension of the anastomosis. Intermittent dearterializations were achieved with the use of an implantable occluder as previously described[9]. In short, the hepatoduodenal ligament and all the attachments to the liver were dissected and ligated including the esophageal arterial branch and the right gastric artery. The sleeves of the miniocc luder were sewn together around the hepatic artery in group D, E, F and G and around both the hepatic artery and the portal vein in group C. The silicone tube from the miniocc luder was taken out subcutaneously and fixed behind the neck of the rat. Occlusions were performed by inflating the balloon with about 0.06–0.08 ml saline once a day during 5 days.

Release of aspartate-aminotransferase (ASAT) and alanine-aminotransferase (ALAT) were used as indicators of liver damage and measured separately in another 18 male Sprague-Dawley rats which were divided into 3 groups: $A =$ end-side porto-caval shunt with transient dearterialization for 30 minutes ($n = 6$); $B =$ end-side porto-caval shunt with transient dearterialization for 60 minutes ($n = 6$); $C =$ side-side porto-caval shunt with transient occlusion of both the portal vein and hepatic artery for 30 minutes. Blood samples were taken at 1, 2, 8 and 24 hours after reperfusion. ASAT and ALAT were analyzed according to the committee on enzymes of the Scandinavian Society for clinical chemistry and clinical physiology[12] and compared to ASAT and ALAT in normal rats, $D$ ($n = 6$).

Statistics: Kruskal Wallis and Mann-Whitney $U$ test were used for statistical evaluation of tumour growth delay. Significant differences of ASAT and ALAT were assessed with One-way ANOVA.

### RESULTS

By the 6th day all tumours were visible under the liver capsule as a well defined grey nodule. At the beginning of the treatment the rats appeared disturbed when the balloon was inflated and deflated but they soon became accustomed to the procedure and occlusions were well tolerated. Tumour volumes and body weights before and after treatment are shown in Table 1. Body weights did not differ significantly between the various treatment groups including sham treated controls ($p = 0.187$ Kruskal Wallis test).

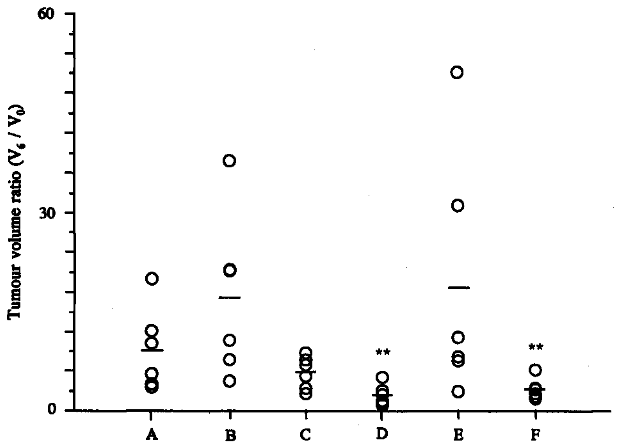

Figure 1 Tumour volume ratio ($V_6/V_0$) after 5 days of treatment. $A$ = control; $B$ = deviation of portal blood flow alone; $C$ = repeated total devascularizations for 30 minutes with a side-side porto-caval shunt; $D$ = repeated dearterializations for 30 minutes with an end-side portocaval shunt; $E$ = repeated dearterializations for 30 minutes and $F$ = repeated dearterializations for 60 minutes alone. $^{**}p < 0.01$ compared with $A$. Values are median and range.

Figure 1 shows differences in tumour growth among groups. Treatments had a significant effect on tumour growth ($p = 0.0012$ Kruskal Wallis test), but portal deviation alone (group B) did not influence the tumour growth as compared with no treatment ($p = 0.089$), indicating a non-significant contribution by portal blood to the growth of the tumours. If an end-side porto-caval shunt was combined with repeated dearterializations for 30 minutes (group D) tumour growth was significantly delayed in comparison with no treatment ($p = 0.004$). Daily dearterializations for 60 minutes in rats having an end-side porto-caval shunt effectively retarded the tumour growth (group E) as shown in Table 1; however, all the rats died by the 4th day due to severe liver damage. Repeated dearterializations for 60 minutes alone was equally efficient in retarding tumour growth as an end-side porto-caval

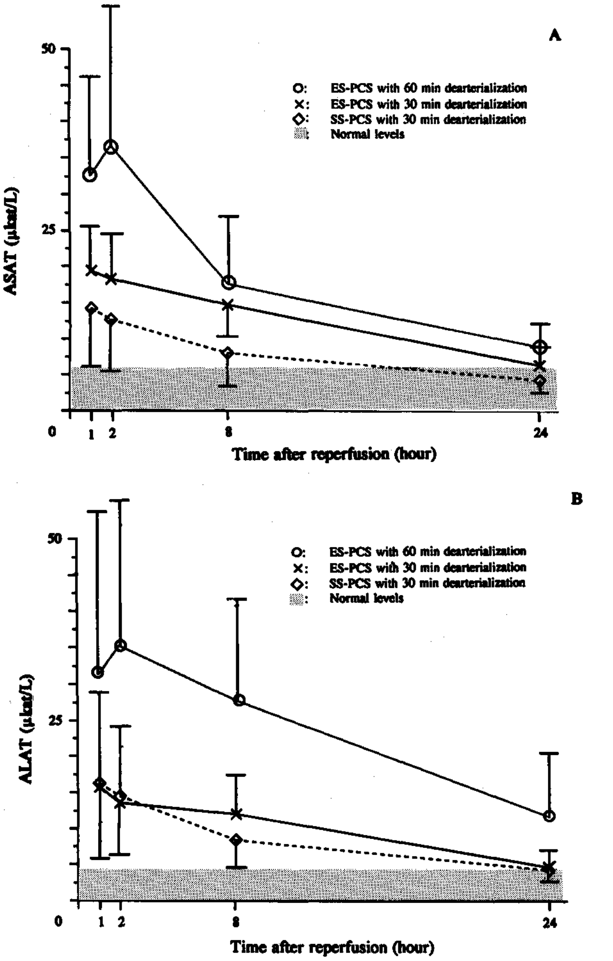

Figure 2A–2B ASAT (2A) and ALAT (2B) at different time periods after reperfusion (mean ± SEM). ASAT and ALAT were released immediately ($p < 0.05$) after reperfusion and showed a tendency to recover in porto-caval shunt rats dearterialized for 30 min. They had returned to normal 24 hours after reperfusion ($p > 0.05$). Whereas, the maximum release of ASAT and ALAT in rats with an end-side porto-caval shunt subjected to 60 min of dearterialization was at 2 hours and they did not restore to normal even 24 hours after reperfusion ($p < 0.05$).

Table 1 Tumour volumes and body weights before and after 5 days of treatment (mean ± SEM).

| | *A* | *B* | *C* | *D* | *E** | *F* | *G* |
|---|---|---|---|---|---|---|---|
| Tumour volume ($mm^2$) | | | | | | | |
| Pre-treatment | 25.00 ± 12.63 | 15.29 ± 5.29 | 16.87 ± 2.82 | 17.27 ± 6.77 | 9.72 ± 4.53 | 39.71 ± 11.87 | 50.81 ± 21.97 |
| Post-treatment | 147.72 ± 49.84 | 140.04 ± 27.84 | 84.77 ± 7.23 | 38.15 ± 8.39 | 6.93 ± 2.75 | 114.49 ± 39.59 | 394.31 ± 65.09 |
| Body weight (g) | | | | | | | |
| Pre-treatment | 288.67 ± 12.22 | 294.66 ± 5.30 | 283.16 ± 9.25 | 269.16 ± 12.78 | 286.75 ± 19.38 | 270.66 ± 10.23 | 299.33 ± 4.74 |
| Post-treatment | 277.33 ± 11.40 | 274.66 ± 4.82 | 251.50 ± 10.50 | 242.33 ± 10.78 | 256.75 ± 22.78 | 245.33 ± 11.58 | 271.67 ± 6.37 |

A: Control; B: Deviation of portal blood flow; C: Repeated simultaneous occlusions of both the hepatic artery and the portal vein for 30 minutes with side-side porto-caval shunt; D: Repeated dearterializations for 30 minutes with an end-side porto-caval shunt; E: Repeated dearterializations for 60 minutes in rats with an end-side porto-caval shunt (all the rats died by the 4th day; *: tumour volumes and body weights at death of the rats; F: Repeated dearterializations for 30 minutes; G: Repeated dearterializations for 60 minutes.

shunt with dearterializations for 30 minutes ($p = 0.007$ vs. control). A side-side porto-caval shunt combined with occlusions of both the portal vein and the hepatic arterial circulation for 30 minutes was not as effective as end-side porto-caval shunt to retard the tumour growth ($p = 0.154$ vs. control). Tumour growth seemed to be increased following intermittent dearterializations for 30 minutes alone, but the growth rate was not significantly higher than no treatment ($p = 0.294$).

Figure 2 shows the release of aminotransferases after different periods of total vascular inflow occlusion. End-side porto-caval shunt with arterial occlusion and side-side porto-caval shunt with occlusion of both the artery and the portal vein for 30 minutes produced a maximum release of ASAT and ALAT within 1 hour after reperfusion, but gradually normalized within 24 hours. Extending the vascular obstructions to 60 minutes in rats having an end-side porto-caval shunt resulted in a progressive release of ASAT and ALAT that did not normalize before the next dearterialization started. All the animals in this group died within 4 days indicating severe liver damage.

### DISCUSSION

This study has shown that deviation of the portal blood by a portocaval shunt did not reduce the growth of an implanted liver tumour. However, by the addition of repeated dearterializations for 30 minutes the tumour growth was significantly retarded. Extending dearterializations to 60 minutes further reduced tumour growth but all rats died after four treatments. On the other hand, repeated dearterializations for 60 minutes alone without deviation of the portal blood was as efficient in retarding the tumour growth as repeated dearterializations for 30 minutes in combination with portal deviation. Repeated normothermic total ischemia for 30 minutes gave rise to a reversible liver injury but extending the ischemic time to 60 minutes caused progressive liver damage.

Responses of liver tumours to intermittent dearterialization have been demonstrated experimentally and clinically[8,9,13–16], though the role of the portal contribution to the tumour during dearterialization is still unsolved. It has been demonstrated that massive tumours have a wide variety of vascular patterns: predominantly arterial, predominantly portal or a mixture of both systems[17,18]. On the other hand the portal supply to liver tumours changes during obstruction of the hepatic artery. A significant increase in portal flow following HAL has been shown to occur both in experimental animals and in patients with liver tumours[7,19,20]. Moreover, two patients with an obstructed portal vein responded to repeat dearterializations with full remission in one patient and partial regression of tumour size and α-fetoprotein in another patient[8]. This might indicate that portal blood, although it does not alter tumour growth when applied alone, may to some extent play a role during dearterialization. As revealed in this study, tumour growth was significantly retarded after 5 days of repeat dearterializations for 30 minutes in rats with an end-side porto-caval shunt. Prolonging dearterializations to 60 minutes seemed not to further reduce the tumour as compared with dearterializations for 30 minutes in the rats with a porto-caval shunt. It seemed that repeat dearterializations were potentiated by portal deviation. However, it remains unclear whether tumour regression was due to the antitumour effect of dearterialization combined with portal deviation or cachexia since serious liver injury was induced in all rats following repeat dearterializations in combination with portal deviation. The rats died within 4 days following 60 minutes of daily dearterializations with portal deviation. In contrast, repeat dearterializations alone for 60 minutes was as efficient in retarding tumour growth without any notable injury to normal liver tissues. Thus, the same growth delay as was seen after 30 minutes of repeat dearterializations in combination with portal deviation was achieved with simple repeat-dearterializations for 60 minutes alone. As we have demonstrated before, repeat dearterializations can be performed for as long as 3 or 4 hours without a significant increase of aminotransferases[21]. This means that repeat dearterializations are selectively cytotoxic to the tumours sparing the normal liver parenchyma.

The main conclusion from this study is that portal deviation during dearterialization seems more harmful than beneficial. If performed long enough repeat dearterializations alone are as effective to delay tumour growth, but less injurious to the liver[9]. This knowledge may have clinical implications in patients who have portal vein obstructed by a liver tumour. In these patients dearterialization, when considered, should be delivered as short repeated occlusions.

### REFERENCES

1. Breedis, C. and Young, G. (1986) The blood supply of neoplasms in the liver. *Am J Pathol.*, **30**, 969–977.
2. Archer, S. G., Gray, B. N. (1989) Vascularization of small liver metastases. *Br J Surg*, **76**, 545–548.

3. Lien, W. M. and Ackerman, N. B. (1970) The blood supply of experimental liver metastases. II. A microcirculatory study of the normal and tumour vessels of the liver. *Surgery*, **68**, 334–340.
4. Ridge, J. A., Bading, J. R. and Gelbard, A. S. *et al.* (1987) Perfusion of colorectal hepatic metastases. Relative distribution of flow from hepatic artery and portal vein. *Cancer*, **59**, 1547–1553.
5. Ackerman, N. B. (1986) Experimental studies on the role of the portal circulation in hepatic tumour vascularity. *Cancer*, **58**, 1653–1657.
6. Ackerman, N. B. (1974) The blood supply of experimental liver metastases. IV. Changes in vascularity with increasing tumour growth. *Surgery*, **75**, 589–96.
7. Taylor, R., Bennett, R. and Sherriff, S. (1979) The blood supply of colorectal liver metastases. *Br J Cancer*, **39**, 749–756.
8. Persson, B. G., Jeppsson, B. and Ekberg, H. *et al.* (1990) Repeated dearterialization of hepatic tumours with an implantable occluder. *Cancer*, **66**, 1139–1146.
9. Wang, L. Q., Persson, B. G. and Bengmark, S. (1994) Repeated dearterializtions of an experimental liver tumour: short and long-term results. *J Surg Res*, **56**, 53–59.
10. Radnell, M., Jeppsson, B. and Bengmark, S. (1990) A technique for isolated liver perfusion in the rat survival and results of cytotoxic drug perfusion on liver tumour growth. *J Surg Res*, **49**, 394–399.
11. Carlsson, G., Gullberg, B. and Gafström, L. (1983) Estimation of liver tumour volume using different formulas—An experimental study in Rats. *J Cancer Res Clin Oncol*, **105**, 20–23.
12. The Committee on Enzymes of the Scandinavian Society for Clinical Chemistry and Clinical Physiology. (1974) Recommended methods for the determination of four enzymes in blood. *Scand J Clin Lab Invest.*, **33**, 291–306.
13. Bengmark, S., Nobin, A. and Jeppsson, B. *et al.* (1983) Transient repeated dearterialization combined with intra-arterial infusion of oncolytic drugs in the treatment of liver tumours. *Recent Results in Cancer Research*, **86**, 68–74.
14. Bengmark, S., Jeppsson, B. and Lunderquist, A. *et al.* (1988) Tumour calcification following repeated hepatic de-arterialization in patients: a preliminary communication. *Br J Surg.* **75**, 525–526.
15. Dahl, E., Fredlund, P. E. and Tylén, U. *et al.* (1981) Transient hepatic dearterialization followed by regional intra-arterial 5-fluorouracil infusion as treatment for liver tumours. *Ann Surg*, **193**, 82–88.
16. Persson, B. G., Nobin, A. and Ahrén, B. *et al.* (1989) Repeated hepatic ischemia as a treatment for carcinoid liver metastases. *World J Surg*, **13**, 307–312.
17. Ackerman, N. B. (1974) The blood supply of experimental liver metastases. IV. Changes in vascularity with increasing tumour growth. *Surgery*, **75**, 589–596.
18. Ackerman, N. B. (1986) Experimental studies on the role of the portal circulation in hepatic tumour vascularity. *Cancer*, **58**, 1653–1657.
19. Jenkins, S. A., Day, D. W. and Mooney, B. *et al.* (1984) The effect of vasopressin and hepatic artery ligation on the blood supply to normal and metastatic liver tissue. *Br J Cancer.* **50**, 785–791.
20. Cagol, P. P., Pian, P. P. D. and Merkel, C. *et al.* (1991) Vascularization of experimental liver metastases. 3-The effect of hepatic artery ligation on blood flow. *J Exp Clin Cancer Res*, **10**, 177–180.
21. Wang, L. Q., Persson, B. G., and Bengmark, S. (1992) Collateral formation after repeated transient dearterialization of the rat liver. In press, *HPB Surg.* **6**, 105–113.

